# Genes-first and phenotypes-first paths to treatment resistance in hematological malignancies

**DOI:** 10.1038/s41419-025-08127-5

**Published:** 2025-11-10

**Authors:** Edoardo Tamellini, Cristina Frusteri, Isacco Ferrarini

**Affiliations:** Department of Engineering for Innovation Medicine, University of Verona, Verona, Italy

**Keywords:** Leukaemia, Haematological cancer

## Abstract

Despite the outstanding achievements of precision medicine in hematology, many targeted therapies eventually fail due to the emergence of resistance mechanisms. Traditionally, a genocentric approach has been adopted to uncover the molecular underpinnings of treatment resistance. This has contributed to identifying resistance gene mutations and designing novel therapeutic molecules with increased potency for the mutant target. However, over the last five years, additional non-genetic adaptations have become increasingly recognized as crucial promoters of treatment resistance. In parallel, emerging works in the field of evolutionary biology suggest that advantageous phenotypic traits appear most often due to cell-intrinsic phenotypic plasticity and can arise independently of gene mutations. In selected cases, single genetic abnormalities such as those involving *TP53* can prime human cells for plasticity and facilitate phenotypic variability. In this narrative review, we retrace the resistance mechanisms to targeted therapies in the framework of these novel evolutionary concepts. We highlight the dichotomy between *genes-first* and *phenotypes-first* pathways of treatment adaptation, with the former being driven by traditional single-point mutations and the latter initiated by the phenotypic diversity and the high-level plasticity of cancer cells. Focusing on resistance mechanisms to kinase inhibitors and BH3 mimetics in leukemias and lymphomas, we describe how each drug can trigger both escape routes, which may even coexist within the tumor bulk of individual patients. Lastly, we provide a three-step translational perspective on how to counteract phenotypes-first resistance mechanisms, with the aim of prolonging disease control in hematological malignancies.

## Facts

- Resistance develops over time in patients affected by hematological malignancies treated with targeted agents.
- Mutations of drug targets have traditionally been considered the mainstay of treatment resistance.
- Non-genetic, phenotypic reprogramming is being increasingly recognized as a key component of drug resistance.
- Initial phenotypic heterogeneity and drug-induced plasticity are hallmarks of cancer and may generate phenotypes-first resistance mechanisms.

## Open questions

- When multiple genetic and phenotypic resistance mechanisms are simultaneously detected in individual patients, which type of resistance arises first?
- What precisely gives rise to phenotypes-first resistance mechanisms? Phenotypic heterogeneity, drug-induced plasticity, or both?
- What are the molecular bases for cancer plasticity, and how could we target them?
- Can we prevent the onset of drug resistance, rather than keep chasing fully established resistance mechanisms?

## Introduction

In the classic view of evolutionary biology, the first step to generate a novel trait is the appearance of a new gene mutation. Such genetic event must provide a reproductive advantage to spread over and replace the initial population. This genes-first scenario implies that each genotype generates a unique phenotype and that DNA-level events are the only drivers of the heterogeneity among living species [1]. Recent experimental advances propelled by single-cell transcriptomics suggest an alternative, or at least complementary, perspective whereby the genome no longer plays the first violin in the evolutionary orchestra [1, 2]. Genetically identical cells can fluctuate between different nonheritable cell states, and each cell state is associated with the expression of specific gene modules and related pathways [3]. Rather than a leap from one discrete cell state to another, there seems to be a transcriptional continuum that covers a range of cell states [4, 5]. Each cell can dynamically change its state over time and transit between different phenotypes starting from the same mutational landscape. The diversity of cell states is enhanced by cell-intrinsic epigenetic reprogramming and microenvironmental-driven signaling modifications [4]. Eventually, transcriptionally defined, nonheritable cell states may stabilize over time and become heritable traits due to subsequent genetic or epigenetic changes. This phenotypes-first process is adopted during development and is thought to accelerate evolution, offering rapid opportunities to adapt to environmental challenges [6, 7]. Emerging evidence indicates that similar phenotypes-first adaptive programs can be co-opted by cancer cells to generate intratumor diversity, cope with harsh environments, and survive antineoplastic treatments [8–10].

## Emerging evolutionary concepts at the intersection with cancer biology

Studies on tumorigenesis have demonstrated that the classical multi-step accumulation of genetic aberrations is a simplistic view of how cancer arises, grows, and spreads. Single-cell transcriptomics of tumor masses from engineered lung and pancreatic cancer models suggest that gain-of-function *KRAS* and loss-of-function *TP53* mutations may not be responsible per se for generating the fully malignant phenotype [4, 8, 9, 11, 12]. Rather, these mutations may foster cell plasticity and enable cancer cells to acquire a broad range of phenotypic states, favoring the progression to more aggressive tumors. Recently, a continuum of resistance states has been discovered in ovarian cancer during treatment with increasing doses of Olaparib [5]. Through a stepwise acquisition of epigenetic changes leading to distinct gene expression programs, phenotypically plastic ovarian cancer cells progressively adapt to olaparib treatment and survive even when treated with a high drug dose. Therefore, the novel phenotypes-first view may apply to spontaneous cancer progression, therapy adaptation, and resistance.

In hematological malignancies, the genes-first view has traditionally explained many resistance mechanisms to targeted agents. Gene mutations potentially compromising the binding of the target protein to a specific drug are the first being sought when a resistance mechanism needs to be elucidated. In some cases, such investigations led to ground-breaking discoveries that pushed forward the synthesis of next-generation inhibitors and helped patients overcome therapy resistance [13, 14]. However, in many others, a “mutational-silent” phenotypic resistance has been found, perfectly fitting into the phenotypes-first framework of evolutionary adaptation [15]. In this review, we describe the main genes-first and phenotypes-first resistance mechanisms that hematologists encounter when administering targeted agents with continuous schedules, particularly BCR-ABL1 inhibitors, BTK inhibitors, and BH3 mimetics. We highlight how disease type and drug class seem to bias resistance mechanisms to genes-first or phenotypes-first routes. Then, we dissect clinical patterns and biological underpinnings of resistance to fixed-duration treatments. Lastly, we explore novel possibilities to tackle phenotypes-first adaptations in hematology.

## Resistance to continuous kinase inhibition in chronic myeloid leukemia and chronic lymphocytic leukemia

### Resistance of chronic myeloid leukemia to BCR-ABL1 inhibitors

Chronic myeloid leukemia (CML) is characterized by the *BCR-ABL1* fusion gene, which constitutes the sole genetic abnormality in most newly diagnosed CML patients. The resulting BCR-ABL1 protein works as a constitutively active kinase that entirely drives disease pathogenesis and activates several downstream pathways, including JAK2/STAT5, MAPK, and PI3K/AKT [16, 17]. Imatinib, the first-in-class BCR/ABL1 inhibitor, dramatically changed the natural history of the disease, turning a fatal disease into a treatable condition with a 10-year survival rate of 83.3% [18]. However, 15–20% of patients under imatinib develop resistance over the first 5 years of treatment [18]. More than 60% of patients with acquired resistance to imatinib harbor BCR-ABL1 kinase domain mutation, centering around the phosphate binding loop, the gatekeeper residue, the SH2 contact, and the activation loop. These variants have been shown to impair imatinib binding, eventually favoring the active conformation of the oncoprotein [19]. Second- and third-generation BCR-ABL1 inhibitors, such as dasatinib, nilotinib, and bosutinib, have been synthesized with the aim of binding imatinib-resistant mutant oncoproteins and overcoming these genetic-based resistant mechanisms. In the CML context, characterized by low genomic complexity and a single driver oncogene [19, 20], the pre-existence or the onset of a new genetic event, namely a *BCR-ABL1* kinase domain mutation, provides a reproductive advantage during inhibitor treatment and may represent the first event driving the emergence of resistant clones. Only a minority of patients with acquired resistance to tyrosine kinase inhibitors do not develop *BCR-ABL1* mutations. In such cases, cell plasticity leading to epigenetic or post-translational fluctuations favors the activation of signaling branches downstream of BCR-ABL1, configuring some degree of phenotypes-first resistant mechanism [21, 22]. Despite this, CML cells acquiring non-genetic imatinib resistance mechanisms may still respond to higher-generation tyrosine kinase inhibitors (TKI), suggesting that cell plasticity is constrained around BCR-ABL1 and that the emerging different phenotypes may still rely on the kinase or scaffolding activity of BCR-ABL1 [19].

### Resistance of chronic lymphocytic leukemia to BTK inhibitors

Chronic lymphocytic leukemia (CLL) is the most common indolent lymphoid malignancy featuring more clinical and biological heterogeneity than CML. Despite never being mutated, the intracellular kinase BTK acts as a signaling hub for CLL cells, transducing pro-survival and pro-adhesive signals from B-cell receptors and chemokine receptors. BTK inhibitors have been game-changers in the treatment algorithms of CLL, yet resistance mechanisms invariably develop after prolonged drug exposure [23, 24]. First reports focused on BTK mutations, particularly C481S, as the main drivers of resistance to ibrutinib, the first-in-class BTK inhibitor [25, 26]. More recent case series have demonstrated that mutations of *BTK* and/or *PLCG2*, encoding a downstream kinase that propagates BTK signaling, are associated with acquired resistance to ibrutinib in 57 and 51% of patients, respectively [27, 28]. Acquired resistance to acalabrutinib, a second-generation BTK inhibitor, is also accompanied by the emergence of BTK mutations in up to 66% of patients [29]. However, there is considerable heterogeneity across patients concerning the cancer cell fraction bearing *BTK* mutations. In the acalabrutinib study, the variant allele frequency (VAF) of *BTK* mutations varied from 0.5 to 95.6% [29], suggesting that other, perhaps non-genetic, mechanisms might come into play within the tumor bulk of individual patients.

### Hypothetical evolutionary scenarios at the emergence of BTK inhibitor resistance

**Fig. 1:**
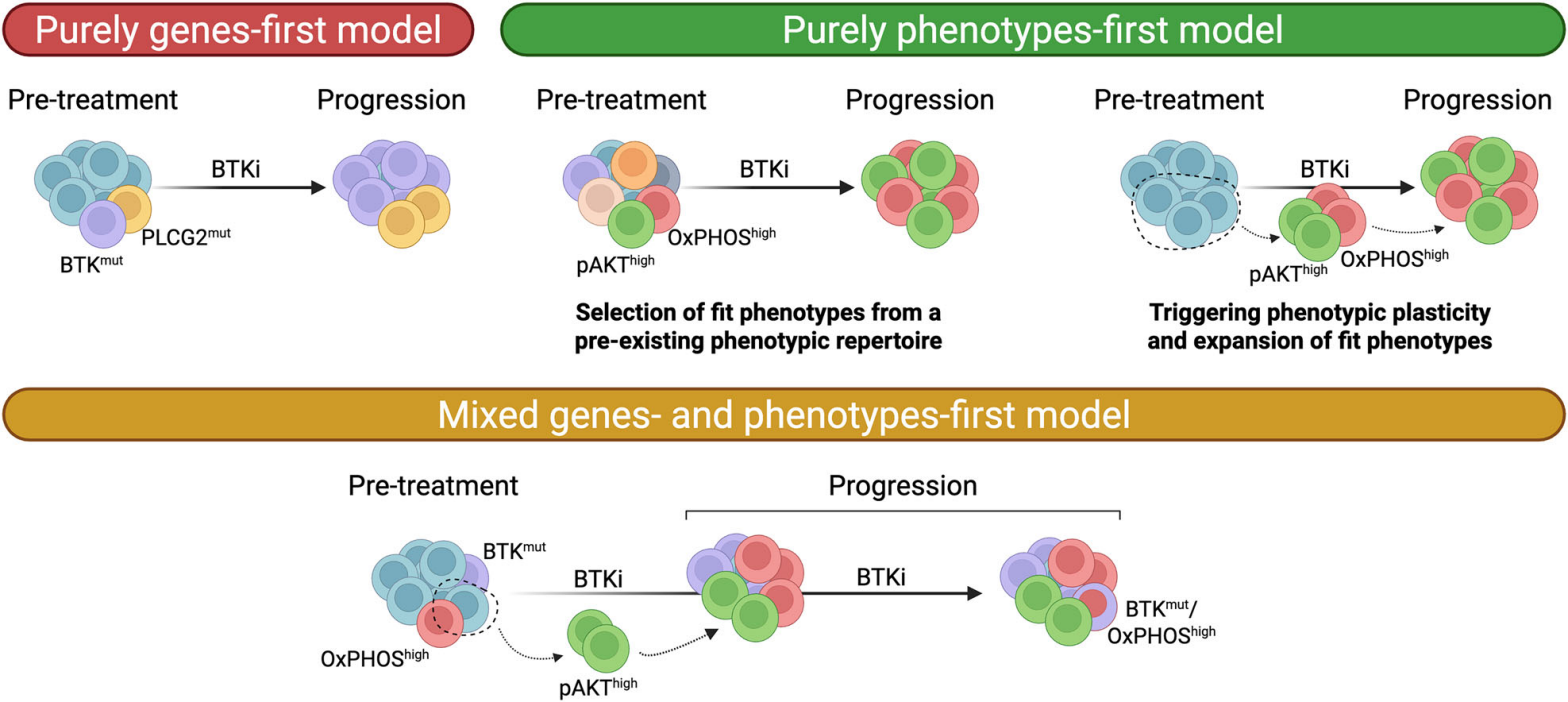
Three different paths to BTK inhibitor resistance in hematological malignancies. In the genes-first model, small-size, pre-existing mutations that confer survival advantage are selected during treatment. In the phenotypes-first model, non-genetic phenotypic cell states that increase cellular fitness, such as those harboring Akt phosphorylation and high OxPHOS activity, are positively selected during treatment. Additionally, tumor-advantageous cell states may arise due to the phenotypic plasticity triggered by anti-cancer treatment. The mixed model contemplates both mechanisms, i.e., resistance genetic mutations that enrich during treatment and the independent appearance of resistant phenotypic states. This phenotypic reservoir of resistant cells may further increase the likelihood to develop genetic resistance. See refs 23–34. BTKi BTK inhibitor, OxPHOS oxidative phosphorylation.

At least three possible scenarios could lie at the basis of resistance heterogeneity to BTK inhibitors (Fig. 1): (i) high VAF patients have evolved a robust genes-first mechanism that fully drives the resistant phenotype, whereas low VAF patients have predominantly transitioned to a range of resistant transcriptional cell states that get stabilized later on by non-mutational, epigenetic changes (*mixed model*); (ii) all patients progress due to the acquisition or expansion of resistance gene mutations, but in a fraction of patients such resistant mutations are yet to be identified (*purely genes-first model*); (iii) all patients progress due to a gradient of phenotypic resistance states that eventually, in some cells, get further stabilized by BTK and other gene mutations *(purely phenotypes-first model)*. Some level of genes-first mechanism has been demonstrated in CLL patients progressing on covalent BTK inhibitors and undergoing treatment with the non-covalent BTK inhibitor pirtobrutinib. In these patients, pre-existing T474x and L528W *BTK* mutations expand rapidly under pirtobrutinib, reaching up to 84% VAF, and are responsible for the resistant status [30–32]. On the other side, a *purely genes-first model* to explain all resistances to BTK inhibitors seems unlikely given the absence of target gene mutations in about 40% of patients and the extensive genomic investigations to which longitudinal CLL samples have been subjected [28]. Mutations other than *BTK* and *PLCG2* in patients progressing on BTK inhibitors have been identified. Still, their recurrence across patients is low, and the VAF in each case is low, making their causal role in disease recurrence unlikely. Therefore, the coexistence of genes-first and phenotypes-first mechanisms in CLL appears more suitable for interpreting the variety of biological findings that have been highlighted in CLL cells re-emerging after continuous BTK inhibition.

## Phenotypes-first adaptation and resistance in B-cell malignancies

### Early phenotypic adaptation to BTK inhibition in CLL

Regardless of the nature of full resistance mechanisms, early non-genetic ibrutinib adaptation through Akt phosphorylation and MAPK pathway activation is likely to play a key role in maintaining a viable CLL cell reservoir. In 70% of ibrutinib-treated patients, the transcription factor FoxO1 is upregulated and promotes the expression of Rictor, an assembly protein for the mTORC2 complex that rapidly phosphorylates Akt. pAkt compensates for BTK inhibition and sustains the survival of CLL cells during early adaptation [33]. An additional study exploiting Assay for Transposase-Accessible Chromatin using sequencing (ATAC-seq) and single-cell transcriptomics in longitudinal CLL samples collected under ibrutinib has demonstrated a large transcriptional and chromatin reprogramming that maintains the MAPK signaling persistently active. This is an example of epigenetic-induced phenotypic plasticity, triggered by a pharmacological intervention. ERK activity, in turn, induced an anti-apoptotic phenotype in persister cells, representing a potential vulnerability [34].

### Non-genetic resistance to BTK inhibition in mantle cell lymphoma and diffuse large B-cell lymphoma

Mantle-cell lymphoma (MCL), a CD5^+^ B-cell malignancy clinically more aggressive than CLL, is unlikely to develop *BTK* mutations during treatment with ibrutinib. A report on 41 MCL patients who experienced disease progression while on ibrutinib has shown that only 17% of them harbored BTK mutations [35]. By contrast, a potentially phenotypes-first transcriptional reprogramming towards oxidative phosphorylation (OxPHOS) and glutaminolysis has been recognized as a driver of ibrutinib resistance in pre-clinical MCL models and patient samples [36, 37]. DNA methyltransferase 3A (DNMT3A) expression is upregulated in resistant MCL cells and induces the expression of MYC target genes, thus contributing to metabolic reprogramming [37]. Likewise, early growth response gene 1 (EGR1) is highly expressed in MCL and diffuse large B-cell lymphoma (DLBCL) cells progressing on ibrutinib and is partially responsible for the increased oxygen consumption rate [38]. These findings suggest that malignant B cells may stochastically fluctuate between OxPHOS^low^ and OxPHOS^high^, two phenotypic states respectively suppressed and selected by therapeutic pressure.

### Resistance to immunomodulatory drugs in multiple myeloma

Non-mutational mechanisms have been also found at the basis of resistance to immunomodulatory drugs (IMiDs) in multiple myeloma (MM). In relapsed MM patients, transcriptional plasticity enables the transcription factor ETV4 to replace IKZF1 (degraded by IMIDs) and to sustain the expression of tumor-promoting MYC and IRF4 [39].

### Resistance to BCR-ABL1 inhibitors in acute lymphoblastic leukemia

In BCR-ABL1^+^ B-cell acute lymphoblastic leukemia (B-ALL), 35% of cases relapsing on TKI harbor resistance mutation of *BCR-ABL1* or gain-of-function mutation of downstream *STAT5A* [40]. An additional 24% of cases progress on TKI due to mutations in the RAS pathway genes, including *KRAS, NRAS*, and *BRAF*, which appear mutually exclusive to ABL1 pathway mutations. About 40% of patients progress through non-genetic, potentially phenotypes-first mechanisms involving a transcriptional shift toward later B-cell developmental stages [40], suggesting that gene mutation-independent lineage plasticity underlies treatment resistance. This is accompanied by the utilization of specific cellular programs, such as pre-BCR signaling, autophagy, and inflammation, which contribute to the resistant phenotype and may represent emerging therapeutic vulnerabilities [40]. In KMT2A-rearranged ALL, lineage plasticity leading to myeloid switch has been demonstrated after immunotherapy. Single-cell epigenome profiling highlighted the coexistence at baseline of ALL subclones with different degrees of plasticity and myeloid potential, which are eventually selected and converted to a fully myeloid program under ALL-directed therapeutic pressure [41]. Thus, tumor-beneficial co-opted developmental programs and pro-survival transcriptional states overlap in leukemic cells, resisting therapeutic pressure and eventually provoking clinical relapse. These and other translational studies support the hypothesis that the more proliferative/aggressive the cancer subtype, the faster the development of resistance by putative phenotypes-first mechanisms [42–44]. Indeed, evidence from evolutionary biology suggests that phenotypes-first processes accelerate evolution [7, 45] and hence might be beneficial for aggressive tumors that are inherently prone to rapidly emerging adaptive solutions.

## The aggressive side of indolent B-cell malignancies: modeling the Waddington landscape of treatment resistance

**Fig. 2:**
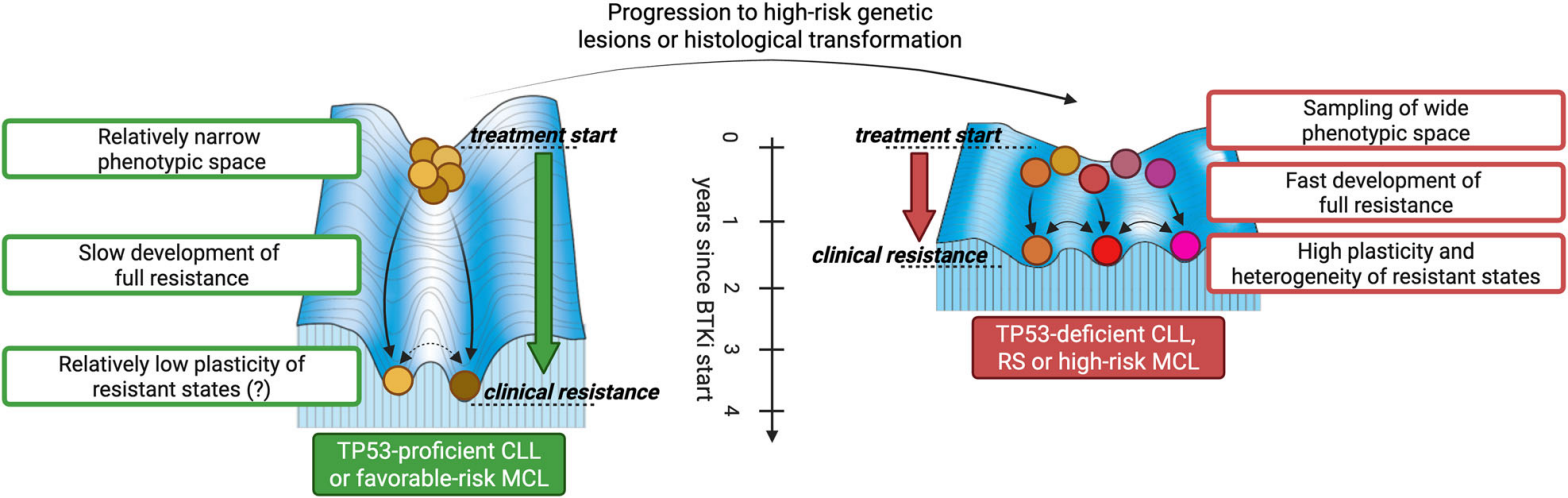
The Waddington landscape of treatment resistance. Low risk CLL and MCL, such as those with wild type *TP53* and no additional unfavorable features, may lie in a relatively narrow phenotypic space with limited variability. In this scenario, an effective treatment such as BTK inhibition generates long-lasting responses, with biological and clinical resistance arising as a late event. Different resistant states may be identified in individual patients, yet the relatively low intrinsic plasticity may limit the phenotypic variability and make these tumors targetable by rational next-line treatments. By contrast, transformation into aggressive histologies or acquisition of plasticity-increasing genetic abnormalities, such as *TP53* aberrations, are associated with higher phenotypic variability at baseline, faster development of multiple resistance mechanisms, shorter duration of clinical response, and possibly high plasticity of resistance states. These are likely to adapt rapidly to next-line treatments by constantly changing their phenotypes or acquiring new resistance mutations. See refs. [46–59]. BTKi BTK inhibitor, CLL chronic lymphocytic leukemia, MCL mantle cell lymphoma, RS Richter syndrome.

A challenging clinical event in the natural history of indolent B-cell malignancies is the transformation into more aggressive histologies. As paradigmatic examples, CLL evolves into Richter syndrome (RS) in about 1 to 10% of cases [46], and classic MCL transforms into blastoid histology in about 35% of patients [47]. The occurrence of transformation usually parallels with a lower overall response and shorter duration of response to BTK inhibitors and other targeted agents. While the median progression-free survival (PFS) of relapsed/refractory (R/R) CLL patients treated with ibrutinib is 51 months [48], two reports show a much lower efficacy in RS [49, 50]. In addition to histological transformation, baseline detection of TP53 aberrations confers inferior PFS to a broad range of cancer therapeutics in CLL and other mature B-cell malignancies. Del[17p] shortens the PFS of CLL patients treated with ibrutinib [51], and similarly, several TP53 abnormalities negatively affect the outcome of MCL patients under BTK inhibition [35]. Overall, these aggressive counterparts do show some response to the targeted agents being used in the indolent phase. However, such responses are less deep and evanish rapidly due to disease progression. Evidence from animal modeling of solid tumors indicates that TP53 abnormalities increase the heterogeneity of transcriptional cell states and foster cell plasticity, enabling cancer cells to sample a wider range of phenotypic space [11, 52, 53]. Borrowing the Waddington developmental landscape [54] to describe treatment resistance, aggressive transformations as well as high-risk indolent phases, both associated with a high frequency of plasticity-increasing genetic alterations, may lie in a more complex and heterogeneous phenotypic space eventually increasing the rate of resistance acquisition (Fig. 2). How much of genes-first or phenotypes-first mechanisms are involved in the generation of such resistance-promoting phenotypic heterogeneity is debatable. High levels of karyotypic complexity and even chromothripsis events, which might directly increase the intratumor phenotypic variability, are often observed in high-risk CLL, MCL, and RS [55, 56]. However, the worst impact on patient prognosis comes when complex karyotype is combined with TP53 aberrations [57–59]. Thus, we speculate that both *phenotypic heterogeneity*, directly following genomic instability, and *phenotypic plasticity*, arising from mutant *TP53*, could play crucial roles in acquired resistance to hematological treatments. The former is a fully fledged genes-first mechanism in which different, unstable subclones give rise directly to as many intratumor phenotypes. The latter appears instead as a phenotypes-first mechanism where the initial gene abnormality is not responsible per se for the resistant phenotype, yet it lays the groundwork for developing phenotypic diversity and plasticity [1].

## Genetic-based resistance to venetoclax in CLL and acute myeloid leukemia

Venetoclax is a Bcl-2 antagonist that mimics the BH3 domain of pro-apoptotic Bcl-2 family members, thus antagonizing the anti-apoptotic Bcl-2 protein and enabling the activation of the effectors Bak and Bax on the mitochondrial surface of Bcl-2 dependent cancer cells. Venetoclax is clinically effective and commercially available for CLL and acute myeloid leukemia (AML), but genetic and non-genetic resistance mechanisms invariably develop over time (Table 1) [60–67].

**Table 1:** Genetic and non-genetic mechanisms of venetoclax resistance in AML and CLL.

Mechanisms of resistance	Type of resistance (genetic/non-genetic)	Disease (AML/CLL)	References
*BCL2* mutations	Genetic	CLL, AML	[60–64, 66]
*BAX* mutations	Genetic	AML	[65]
Loss[8p]	Genetic	CLL	[64]
*MCL1* copy gain	Genetic	CLL	[15, 64]
NFκB pathway activation	Non-genetic	CLL	[15]
Upregulation of Bcl-xL or Bfl-1	Non-genetic	CLL	[15, 60, 85]
*BBC3* hypermethylation	Non-genetic	CLL	[86]
Hyperphosphorylation of Bcl-2 family	Non-genetic	CLL	[87]
Functional decrease of apoptotic priming	Non-genetic	AML	[89]
ClpB-mediated tightening of mitochondrial cristae	Non-genetic	AML	[73]
Increased contact mitochondria/ER contact	Non-genetic	AML	[90]
Mitophagy	Non-genetic	AML	[90]
Increased OxPHOS activity	Non-genetic	AML	[73, 75]
Increased MCU activity	Non-genetic	AML	[92]
CD40 activation	Non-genetic	CLL	[77]
JAK/STAT signaling	Non-genetic	CLL	[96]
Contact with mesenchymal stromal cells	Non-genetic	AML	[100]
Monocytic escape	Non-genetic	AML	[78, 158]
Megakaryoblast/Erythroid differentiation	Non-genetic	AML	[104]
Lineage plasticity	Non-genetic	AML	[78, 103–105, 107]
RAS mutations	Genetic	AML	[67]
NOTCH1 activation	Non-genetic	CLL	[79]
High IFNγ signaling	Non-genetic	AML	[80, 81]
ABCC1 overexpression	Non-genetic	AML	[91]
RPS6KA1 overexpression	Non-genetic	AML	[82]
ULK1 overexpression	Non-genetic	AML	[83]

### Genetic-based resistance to venetoclax in CLL

The existence of genetic adaptation to venetoclax is suggested by the emergence of *BCL2* mutations under the therapeutic pressure of the drug. Forty-six per cent of venetoclax-treated CLL patients had the G101V *BCL2* mutation, which was not detectable prior to venetoclax initiation [60]. This point mutation caused a 180-fold reduction of Bcl-2 affinity for venetoclax, preventing the drug from displacing pro-apoptotic members and ultimately blocking the apoptotic cascade [60]. D103Y *BCL2* mutation was also identified in CLL patients acquiring resistance to venetoclax, similarly affecting the drug-target binding [62]. The proportion of CLL cells bearing one of the *BCL2* mutations varied from 1.4 to 60%, suggesting profound intratumor, other than interpatient, heterogeneity in terms of resistance mechanism [61, 62]. A more recent report on CLL patients with dual resistance to BTK inhibition and Bcl-2 antagonism has identified emerging *BCL2* mutations in 4 out of 11 cases, with only 2 characterized by a mutated cancer cell fraction above 25% [63]. Similarly, Khalsa and colleagues found G101V *BCL2* mutation in 4 out of 11 venetoclax-progressing patients, with a VAF of only 0.03 and 4.68% in 2 of them [64]. In addition, loss[8p] and copy number gain of *MCL1*, an anti-apoptotic member alternative to Bcl-2, have been identified as potentially genes-first mechanisms of acquired venetoclax resistance in CLL [64].

### Genetic-based resistance to venetoclax in AML

At variance with CLL, AML cells adapting to venetoclax treatment are less likely to display *BCL2* mutations [66]. In contrast, AML patients acquire one or more inactivating *BAX* mutations in 17% of cases [65]. AML cells with Bax deficiency resist BH3 mimetics while preserving variable degrees of sensitivity to chemotherapy. Among patients with a *BAX* abnormality, only 12% had a *BAX* VAF of more than 5%, suggesting that, even in the AML setting, multiple resistance mechanisms might coexist within the same patient [65].

### TP53 aberrations at the crossroad between genes-first and phenotypes-first resistance

**Fig. 3:**
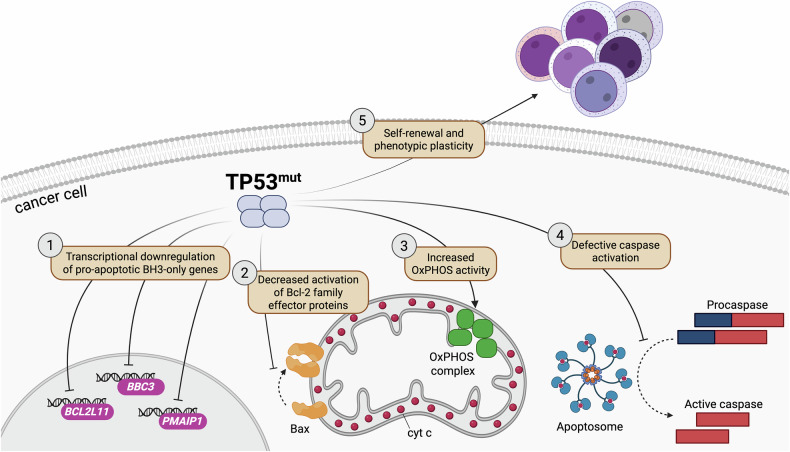
Anti-apoptotic and plasticity-promoting effects of TP53 mutations in cancer. TP53 aberrations cause transcriptional downregulation of pro-apoptotic BH3-only genes, such as *BCL2L11*, *BBC3* and *PMAIP1*, encoding Bim, Puma and Noxa, respectively. This leads to decreased functional activation of effector proteins Bak and Bax under pro-apoptotic conditions. TP53 mutations also impact cellular bioenergetics by increasing OxPHOS activity in AML. Moreover, they counteract cytosolic caspase activation downstream of cyt c release and increase self-renewal and plasticity. See refs. [42, 68–71]. cyt c cytochrome c, OxPHOS oxidative phosphorylation.

Tumor-suppressor TP53 is crucial to maintaining biological homeostasis, particularly mediating cell-cycle arrest, senescence, and apoptosis in damaged cells. Disruption of TP53 occurs in several cancer types, paving the way to a treatment-resistant neoplastic transformation and phenotypic plasticity [42, 68–71]. Most TP53 alterations are loss-of-function missense or deletion mutations, but some could harbor gain-of-function (GOF) activity [72]. The TP53 R175H GOF mutation acts as an oncogene in hematopoietic stem cells (HPSC), driving the leukemic onset. A critical mediator in this process is the embryonic transcriptional factor FOXH1, which is involved in determining the stemness properties of normal HPSC. TP53-R175H enhances FOXH1-driven transcriptional programs, promoting self-renewal and cell plasticity, resulting in leukemic transformation and conferring cancer cells the ability to dynamically adapt their phenotype under virtually any type of therapeutic pressure [42]. Figure 3 illustrates the main mechanisms whereby TP53 mutations suppress pro-apoptotic functions and foster therapy resistance in CLL and AML [69, 70].

## Phenotypes-first paths to venetoclax resistance in CLL and AML

A growing number of studies have pointed out non-genetic mechanisms of cellular adaptation to Bcl-2 antagonism, including mitochondrial remodeling, metabolic rewiring, tumor-beneficial interactions with microenvironment, and lineage plasticity [73–83]. Because their molecular bases do not necessarily encompass genomic aberrations but rather modifications in protein-protein interaction and adaptive changes in the transcriptional state, these mechanisms may be classified as bona fide phenotypes-first cellular attempts to overcome venetoclax therapeutic pressure.

### Multi-level mitochondrial adaptation

CLL patients treated with continuous venetoclax usually experience two waves of non-genetic mitochondrial adaptation. The first wave occurs right after venetoclax initiation and is characterized by the upregulation of anti-apoptotic proteins of the Bcl-2 family, mediated in part by soluble factors such as BAFF [84]. This represents a first line of adaptation that allows a fraction of CLL cells to survive the first weeks of venetoclax ramp-up. The second wave occurs at the emergence of full clinical resistance. Single-cell transcriptomic studies have demonstrated that CLL cells progressing on venetoclax transitioned to an NFκB^high^ state that promoted the transcription of the alternative anti-apoptotic member Mcl-1, which in turn took the lead of cell anti-apoptotic defense at the expense of Bcl-2 [15]. Notably, this mechanism was fully reversible upon venetoclax discontinuation in vivo, in line with its nonheritable, non-genetic nature. In only a minority of patients, Mcl-1 upregulation was stabilized by 1q21 amplification and thus became fully heritable [15]. An additional fraction of relapsing patients developed transcriptional upregulation of other anti-apoptotic members such as Bcl-xL and Bfl-1 [15, 60, 85]. Moreover, in some cases, subclonal genetic loss of the pro-apoptotic *BAX* or the hypermethylation of *BBC3* (encoding Puma) coexisted with the above-mentioned phenotypes-first mechanisms [15, 86].

Post-translational modifications represent an additional layer of Bcl-2 family rearrangement in venetoclax-resistant CLL. A recent report has revealed the phosphorylation of several Bcl-2 family members, including Mcl-1 at threonine 163, in CLL cells resistant to venetoclax [87]. At least some of the phosphorylated forms of the Bcl-2 proteins had more robust anti-apoptotic functions than their native counterparts and were more effective at blocking apoptotic signaling. While in CLL cells the functional apoptotic priming (i.e., the proclivity to undergo mitochondrial apoptosis) remains high even in relapsed/refractory patients [87, 88], in AML relapsing after chemotherapy or venetoclax there is evidence of a functional shift towards a lower apoptotic priming as measured by BH3 profiling and other functional methods [89]. Loss of apoptotic priming compromises the ability of AML mitochondria to release enough cytochrome c to trigger the apoptotic cascade.

ClpB-mediated tightening of the mitochondrial cristae and contact points between the outer mitochondrial membrane and the endoplasmic reticulum are two types of mitochondrial ultrastructural rearrangements potentially involved in decreasing the apoptotic priming of AML cells [73, 90]. In addition, specific metabolic pathways are upregulated by leukemic cells adapting to venetoclax. Elevated glutathione and nicotinamide metabolism has been identified as a metabolic hallmark of venetoclax-resistant AML cells, which in turn promotes amino acid metabolism and fatty acid oxidation, eventually fueling OxPHOS activity [91]. High steady-state levels of mitochondrial calcium, mediated by the mitochondrial calcium uniporter (MCU), have also been detected in resistant cells [92]. Consistently, inhibition of glutathione metabolism, inactivation of nicotinamide phosphoribosyltransferase, and suppression of MCU activity exert selective toxicity on venetoclax-resistant AML cells [75, 91, 92].

In such resistant cells, metabolic rewiring through increased glutathione synthesis and fatty acid oxidation is a regulatory mechanism of cell fate. Transcription factors like ATF4, activated via the integrated stress response, modulate key metabolic genes, fueling resistance. Additionally, mitochondrial metabolism regulators such as PGC-1α have emerged as plasticity modulators by maintaining mitochondrial integrity under stress in cancer cells [93, 94].

### Interactions with microenvironment

In CLL cells, CD40 activation by microenvironmental CD40-ligand expressing cells (such as T-follicular cells or fibroblasts) upregulates Bcl-xL and Mcl-1, thus conferring resistance to Bcl-2 inhibition [77, 95, 96]. On the other hand, the downregulation of CD40 expression mediated by the inhibition of TLR-9, mTOR1/2, or electron transport chain (ETC) sensitizes CLL cells to venetoclax [97, 98].

In AML, venetoclax has been suggested to increase T-cell anti-leukemic activity by promoting reactive oxygen species production [99]. Furthermore, mesenchymal stromal cells interact with leukemic cells to enhance Mcl-1 expression. Simultaneously, Mcl-1 upregulates surface adhesion molecules responsible for leukemia-stroma interactions, sustaining a venetoclax-resistant anti-apoptotic state [100]. Notably, bone marrow microenvironment promotes early adaptation to other classes of antineoplastic agents, providing AML cells the time to evolve more stable and microenvironment-independent mechanisms [101]. Additionally, a single-cell multiomic analysis combining scRNA-seq, surface proteomics and mutational profiling in AML revealed that CD36^high^ subpopulations display intrinsic resistance to venetoclax and emerge during relapse. These cells did not carry new genetic mutations but had distinct transcriptional and metabolic signatures, making them targetable with fatty acid metabolism inhibitors [102].

### Leukemic stem cell lineage plasticity

Leukemic stem cells (LSC) in AML display a high phenotypic and epigenetic plasticity that represents a drug-escape mechanism. The variety of differentiation states that LSC could take ranges from undifferentiated hematopoietic stem cell-like to more differentiated stages such as myeloid progenitors-like, monocytic-like, megakaryoblast-like, or erythroid-like. Under therapy influence, LSC can navigate into a complex process of differentiation/de-differentiation to acquire new phenotypic characteristics and develop treatment resistance [78, 103]. A venetoclax-resistant LSC with monocytic features characterized by low Bcl-2 dependency has also been described. This sub-population could be predominant in primary refractory disease, but most often represents a minor fraction positively selected by venetoclax treatment, inducing disease relapse after a first remission phase [78]. Similarly, megakaryoblast and erythroid differentiations confer an intrinsic resistance to Bcl-2 inhibition, as the survival of these cells predominantly depends on Bcl-xL and Mcl-1 [104]. Integrating single-cell transcriptomics and ex vivo drug testing, four different venetoclax-resistant clusters have been characterized. Notably, one of them harbors transcriptional and phenotypic features of monocytic and dendritic blasts, and another one shows footprints of erythroid differentiation [105]. This further supports that lineage plasticity is involved in tuning sensitivity to Bcl-2 antagonism. Moreover, four LSC types with distinct phenotypic properties have been identified. Notably, the LSC type LMPP (Lymphoid Myeloid Primed Progenitor) could lose Bcl-2 dependency under venetoclax treatment and move toward Bcl-xL-dependency [103].

Comparable resistance mechanisms have been proven for menin inhibitors in KMT2A-mutated AML. Following the transition into a monocytic state, leukemic cells rely on scaffold proteins other than menin to bind chromatin, compromising treatment efficacy [106].

Lastly, single-cell RNA sequencing studies comparing pediatric AML at initial diagnosis and relapse have demonstrated a significant difference in lineage composition. At relapse, the cell population was characterized by the under-representation of myeloid transcriptional programs in favor of other lineages, including undifferentiated and non-myeloid states, such as B-lymphoid-like progenitors [107].

## Additional insights into phenotypic plasticity in AML

Recent breakthroughs using high-resolution single-cell and lineage-tracing technologies have provided evidence that phenotypic resistance in AML is not merely inferred but empirically trackable across treatment courses. scATAC-seq of therapy-resistant AML has shown that malignant cells span a diverse lineage spectrum, including HPSC-like, myeloid, erythroid, and lymphoid-primed states. These epigenetically poised states persist through therapy and are not predicted by mutational burden, reinforcing a model of epigenetically driven phenotypic plasticity [108]. Moreover, transcriptional and chromatin accessibility profiling across diagnosis, remission, and relapse stages in pediatric AML revealed therapy-induced lineage switching. In relapsed samples, cellular hierarchies transitioned toward a more primitive state. Such primitive cells harbored an over-representation of non-myeloid lineage programs, with the appearance in some patients of a B-lymphoid-like hierarchy [107]. More recently, Saxe and colleagues developed the FLARE (Following Lineage Adaptation and Resistance Evolution) lineage-tracing platform, which maps the evolution of cancer cells under the selective treatment pressure. Applying this technique to AML cells exposed to cytarabine, they uncovered a broad spectrum of resistance signatures attributed to diverse cell transcriptional changes [109].

## Distinguishing pre-existing versus therapy-induced plasticity

Phenotypic plasticity in hematologic malignancies can arise from two coexisting but mechanistically distinct processes: pre-existing cell-state heterogeneity and therapy-induced transcriptional reprogramming. Most discussion from the previous sections concerns therapy-induced plasticity. However, there are examples of pre-existing transcriptional plasticity. Single-cell transcriptomic studies in DLBCL provide compelling evidence for pre-existing plasticity. Roider et al. demonstrated that individual DLBCL tumors comprise multiple transcriptionally distinct malignant subpopulations, even prior to therapy. These subpopulations varied in proliferative, metabolic, and immune-interactive gene expression programs, including differences in MHC-II expression and MYC signaling activity. Importantly, these subtypes correlated with ex vivo drug sensitivities but not with genetic alterations, indicating that transcriptional plasticity and functional diversity exist independently of clonal mutations [110]. Further extending this insight, malignant states persist across spatial regions within a single lymph node and across involved anatomical sites. Lineage inflection and developmental imprinting, such as plasmablastic versus centroblastic programs, were evident across clones, mimicking normal B-cell ontogeny and demonstrating that transcriptional plasticity reflects co-opted developmental hierarchies [111]. Notably, transcriptional plasticity in DLBCL does not map cleanly onto genetic heterogeneity, underscoring that non-genetic mechanisms are primary drivers of phenotypic diversity in these tumors [111].

Clinically, the coexistence of intrinsic and adaptive plasticity implies that early therapeutic interventions should address the full landscape of pre-existing states (e.g., through combination therapies), while longitudinal treatment should anticipate and counteract reprogramming dynamics.

## Is it time to combine genetic and functional profiling in the clinic?

While the predictive role of functional/phenotypic profiling in clinical practice is still limited, it is likely that the combination of genetic and functional/phenotypic testing will improve prognostication and refine treatment assignation in the next future. Regarding the functional/phenotypic side, an important point is whether we should profile on a patient-by-patient basis or on a disease-by-disease basis. Two examples of the former scenario are the flow cytometry profiling of anti-apoptotic proteins in AML [112] and the flow cytometry assessment of the leukemic stem cell lineage [78]. Such phenotypic features differ on a patient-by-patient basis and, importantly, they complement the mutational assessment and help predict sensitivity to Bcl-2 antagonism in AML. An example of the latter is the phosphorylation profiling of CLL and MCL under ibrutinib therapeutic pressure [33, 113]. The phosphorylation of Akt can be considered a general feature of CLL and MCL cells adapting to ibrutinib. In this case, there should be no need to profile each individual patient, and drugs targeting the Akt pathway could be proposed to suppress the emergence of ibrutinib resistance in the majority of these patients.

**Fig. 4:**
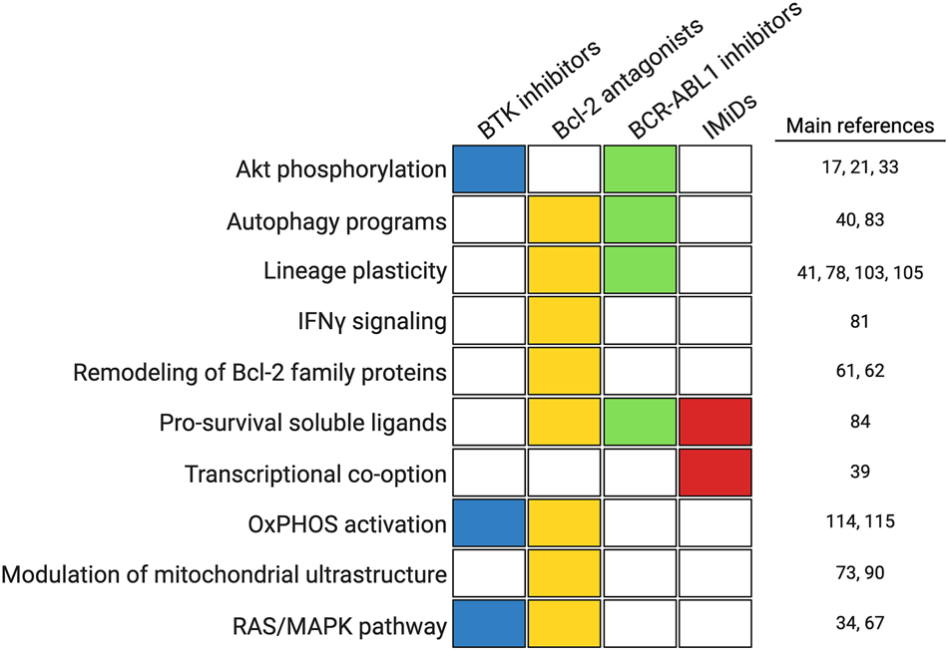
Non-genetic resistance mechanisms to four different drug classes. The heatmap illustrates functional pathways underlying non-genetic adaptation and resistance to BTK inhibitors, Bcl-2 antagonists, BCR-ABL1 inhibitors, and immunomodulatory drugs (IMiDs). While some mechanisms confer resistance to one specific drug class, others are more promiscuous and can undermine sensitivity to multiple types of therapeutic agents. Main references are listed on the right. OxPHOS oxidative phosphorylation.

An additional question relates to the extent to which non-genetic adaptations are shared across diverse drug classes. To provide a definitive answer, primary tumor cells collected from different patients that are adapting to different drug classes should be profiled with the same technique. To the best of our knowledge, no such study has been conducted in hematology. What has been done so far is studying treatment adaptation to one drug at a time, often using techniques that explore only selected aspects of cell biology (mRNA expression, post-translational modifications, organellar biology, etc.). With this fundamental limitation in mind, Fig. 4 illustrates non-genetic resistance mechanisms to different drug classes (BTK inhibitors, venetoclax, BCR-ABL1 inhibitors, IMiDs). Table 2 reports selected in vivo studies validating the existence of phenotypes-first resistance mechanisms and providing the rationale to design phenotypes-directed therapeutic strategies [114–121]. In Table 3, we have selected three clinical scenarios in which understanding phenotypic plasticity helped inform salvage therapeutic strategies [88, 122, 123].

**Table 2:** Selected animal studies and clinical trials supporting phenotypes-first resistance mechanisms in hematological malignancies.

Disease	Model System	Phenotypic mechanism of resistance/sensitivity	Key Observations	Reference
Acute myeloid leukemia	PDXs, clinical trials	Metabolic reprogramming (OxPHOS ↑, Mcl-1 ↑)	Targeting OxPHOS could overcome venetoclax resistance; induction of mitochondrial stress could restore venetoclax efficacy	[114–116]
Acute myeloid leukemia	Clinical trials	Epigenetic reprogramming	hypomethylating agents could enhance immunotherapy activity by upregulating PD-L1	[117]
Acute lymphoblastic leukemia	PDXs, CAR-T trials	Antigen escape via splicing / epigenetic suppression of CD19	CD19-negative relapse without mutation; reversible silencing	[118]
Chronic myeloid leukemia	Clinical trials (ENESTfreedom)	Drug holiday	Relapse after TKI cessation involves same clone re-expanding without new mutations	[119]
Myelodysplastic syndromes	Clinical samples analysis	Epigenetic resistance (hypermethylation)	Aberrant hypermethylation is an independent prognostic predictor for survival	[120]
Multiple Myeloma	PDXs	Microenviromental interaction	CXCR4 inhibitor sensitizes myeloma cells to therapeutic agents	[121]

**Table 3:** Three anecdotal patients who benefitted from the identification of non-genetic resistance mechanisms.

Disease	Description	Reference
Acute myeloid leukemia (AML)	In this case study of relapsed/refractory FLT3-wild-type AML, resistance to venetoclax was not due to BCL2 mutation but associated with elevated MCL-1 expression. Preclinical modeling revealed that combining gilteritinib with venetoclax suppressed MCL-1 and restored sensitivity. This combination achieved clinical tumor reduction and enabled successful bridging to transplant, illustrating how targeting phenotypic resistance via MCL-1 modulation can be clinically actionable.	[122]
Richter syndrome	Here is described a patient who developed a venetoclax-emergent Richter transformation. Functional profiling revealed that transformed cells were highly MCL-1 dependent partly due to elevated BTK phosphorylation. Treatment with ibrutinib led to MCL-1 downregulation and induced rapid cell death, resulting in clinical improvement.	[88]
Chronic lymphocytic leukemia (CLL)	Idelalisib-resistant B-cell malignancy models showed high dependency upon the proteasome degradation system. A patient with multi-drug resistant CLL showed clinical improvement following treatment with proteasome inhibitor and Bcl-2 antagonist.	[123]

## Multiple relapsed CLL patients treated with novel BTK-directed agents: an opportunity to gather new insight on resistance to continuous treatments

A number of factors hamper the acquisition of new knowledge about the onset and propagation of resistance mechanisms. First, in several hematological malignancies, the leukemic component is only a small fraction of the tumor bulk, with the majority of cells residing in less accessible niches such as lymph nodes and bone marrow. Second, many drugs (chemotherapy, BH3 mimetics) have a rapid and direct pro-apoptotic effect on circulating cells. Thus, the leukemic fraction is the first that disappears after treatment and, in most cases, the last to re-appear upon resistance development. Third, in leukemic B-cell malignancies treated with lymphocytosis-inducing drugs (i.e., BTK inhibitors) clinical responses are usually so durable that studying stepwise genetic and phenotypic adaptation and resistance would require longitudinal sample collections over many years.

**Fig. 5:**
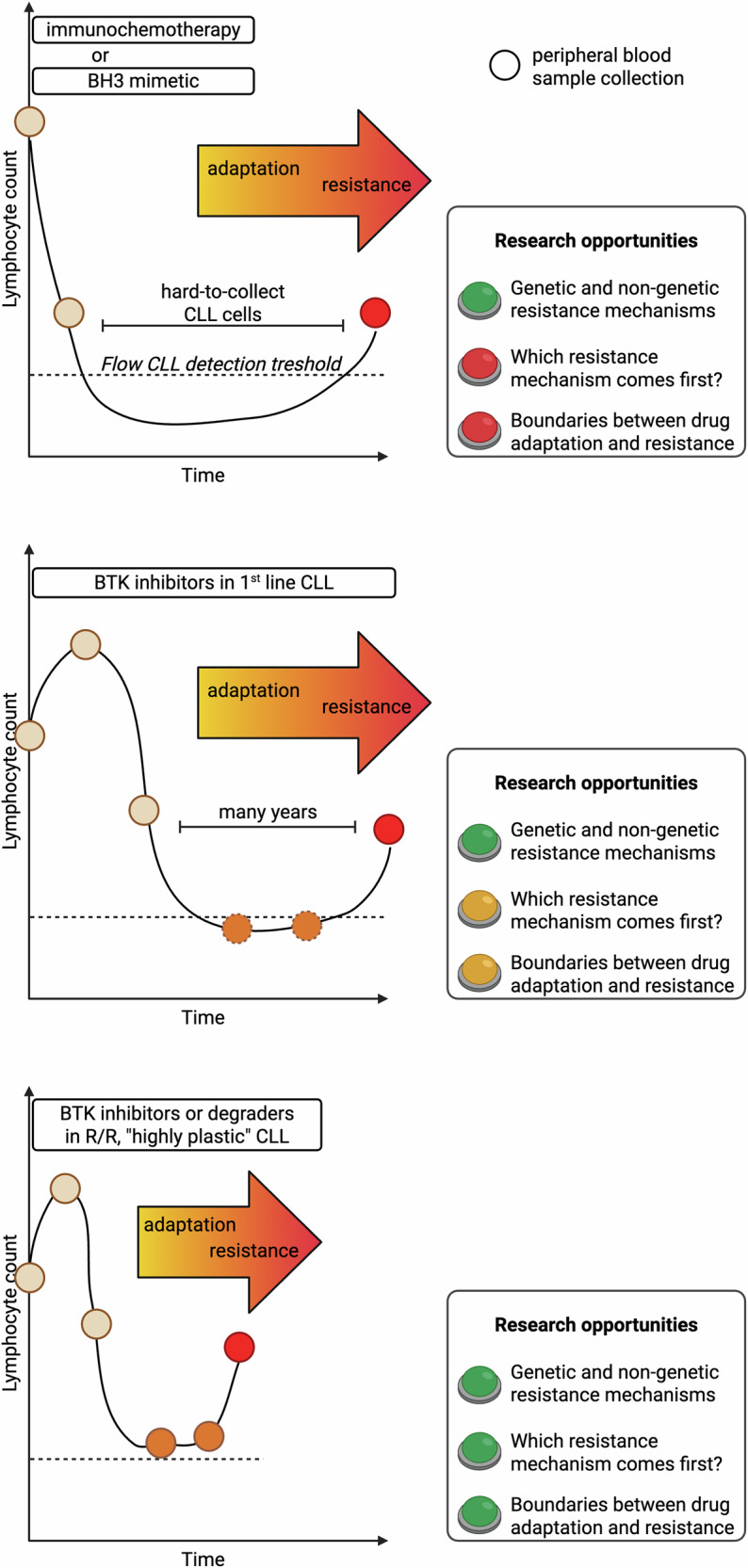
Different therapeutic regimens and different CLL patient populations offer diverse research opportunities to advance our understanding of adaptation and resistance. *Upper panel*. Immunochemotherapy and BH3 mimetics have direct pro-apoptotic effect on leukemic CLL cells, causing a rapid decrease of peripheral blood malignant lymphocytes. In the following months to years, CLL cells are undetectable in peripheral blood; if detectable, they are too few for extensive ex vivo investigations. Reappearance of lymphocytosis usually occurs when full resistance (genetic, non-genetic, or both) has already developed. In this scenario, only the comparison between pre-treatment and fully resistant samples is feasible. *Middle panel*. First or second generation BTK inhibitors cause the redistribution of CLL cells, with the shrinkage of lymph nodes and the subsequent increase of leukemic cells. After the first weeks to months, circulating CLL cells progressively die and may become barely detectable (dashed circles). If these drugs are given as first-line treatment, their efficacy is so durable that it takes many years for adaptation and resistance to develop. This poses significant experimental challenges due to the prolonged treatment timeframe and the high number of samples that should be collected longitudinally. *Lower panel*. When BTK-directed agents are given to multiple relapsed/refractory (R/R) CLL patients, inherently characterized by higher plasticity compared to treatment-naïve patients, leukemic cells remain more easily detectable in the peripheral blood during treatment (personal observations). Moreover, adaptive and resistance mechanisms develop more rapidly after treatment initiation. In this context, longitudinal collection of peripheral blood samples is feasible and may provide fresh insights on the stepwise acquisition of adaptive and resistance mechanisms. See refs. [124–127].

Clinical studies enrolling multiple relapsed CLL patients, who are inherently prone to develop resistance to the next-line therapy, and testing the activity of novel lymphocytosis-inducing BTK-directed treatments, i.e., non-covalent inhibitors and protein degraders [124, 125], now offer a unique opportunity to explore new frontiers of drug adaptation and resistance in a shortened timeframe (Fig. 5). In this context, the application of novel methods such as genotyping of transcriptomes (GoT), which couples the detection of gene mutations with the analysis of transcriptome at the single cell level, may help identify which resistance mechanism comes first and how much the cellular transcriptome differs between genetically and phenotypically resistant clones [126, 127]. Overall, the exploitation of integrated single-cell approaches in leukemic cells collected during the adaptation phase (right before the emergence of full resistance) might reveal insights on programs utilized by leukemic cells to persist under drug pressure and to switch from “silent” adaptation to full resistance acquisition. This could help detect plasticity-driven clones early on and find new strategies to prevent their expansion.

## Focus on fixed-duration treatments: when resistance emerges despite the stop of therapeutic pressure

**Fig. 6:**
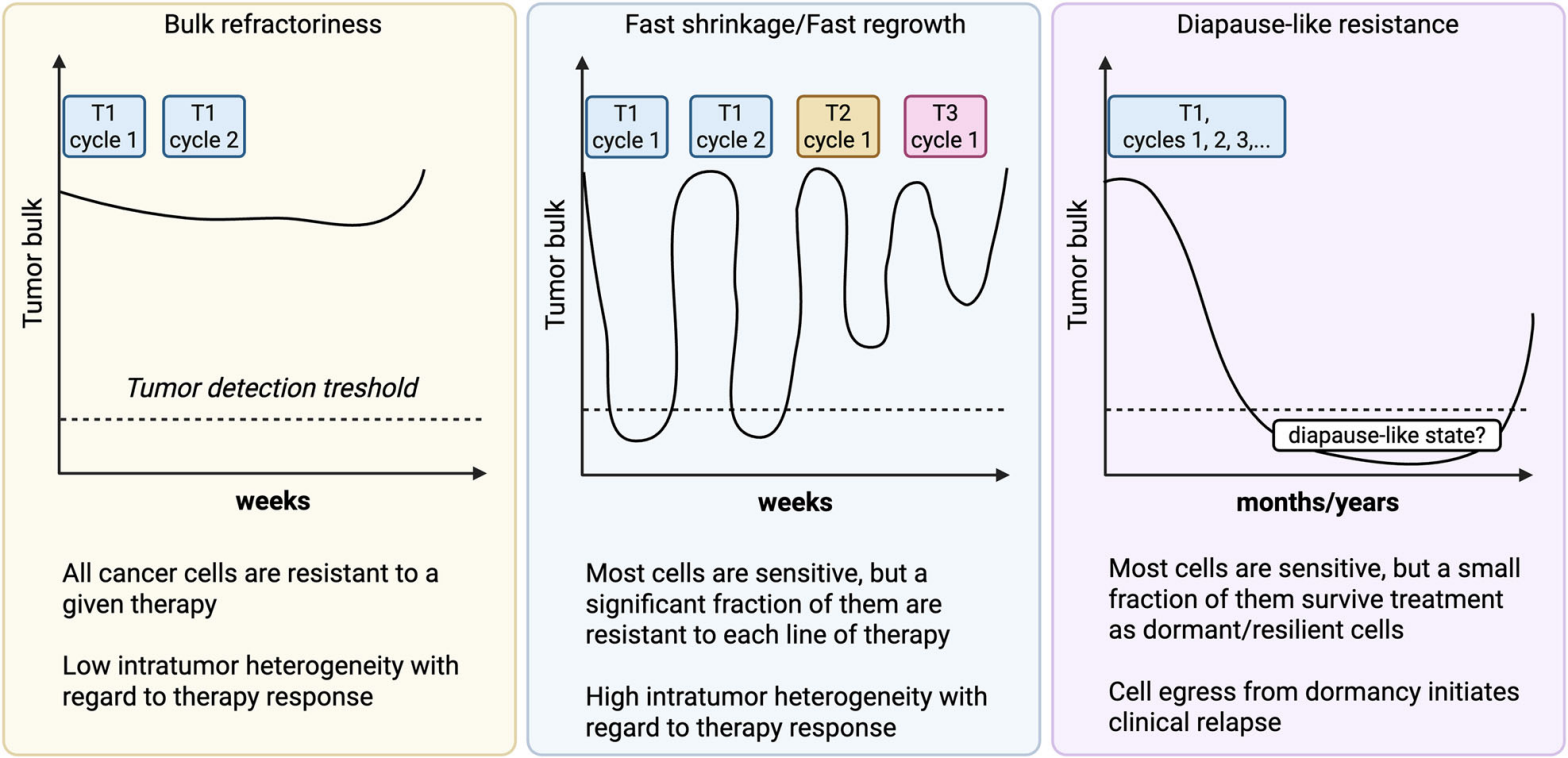
Three distinct patterns of clinical resistance to fixed-duration treatments (e.g., chemoimmunotherapy) in hematological malignancies. *Left panel*. ***Bulk refractoriness*** is characterized by a substantial lack of sensitivity to a given therapy. The majority of cancer cells survive treatment and tumor masses keep expanding. Such clinical behavior suggests a low intratumor heterogeneity with regards to therapy response, as all cells are concordantly unresponsive. *Middle panel*. ***Fast shrinkage/fast regrowth pattern*** is more typical of the most aggressive non-Hodgkin lymphomas (e.g., high-grade B-cell lymphomas) and is characterized by rapid response to treatment and just as rapid (within days) tumor regrowth. This behavior often recurs with consecutive treatments until therapeutic options expire. Despite the lack of basic studies in the field, we speculate that these tumors share high intratumor heterogeneity (i.e., they are highly plastic) with regard to therapy response: most cells are killed, yet a significant fraction persists and rapidly repopulates the original tumor. Due to the high tumor-intrinsic plasticity, regrowth cells will be heterogeneous as well in terms of therapy response, and resistance cycles will proceed the same way for a variety of subsequent therapies. *Right panel*. In ***Diapause-like resistance***, the tumor bulk responds to treatment but a small fraction of cancer cells survives for months to years as dormant or resilient cells. Dormant cells share a diapause-like transcriptional program (see text and refs. [128–133]). Egress from such cell programs initiates clinical relapse. T1, T2, T3: different types of therapy.

Although fixed-duration regimens (including chemotherapy and venetoclax-based combinations) are deemed effective in preventing genetic and non-genetic adaptation due to the early stop of therapeutic pressure, they can be hampered by three distinct patterns of clinical resistance, each with possibly different biological underpinnings (Fig. 6). Among these patterns, *diapause-like resistance* is being increasingly characterized at the molecular level in solid and hematological malignancies.

### Diapause-like resistance programs in solid and hematological malignancies

In solid tumors, cells persisting after chemotherapy enter a quiescent state that transcriptionally resembles embryonic diapause, a stage of suspended development triggered by stress and associated with attenuated MYC and enhanced cyclin-dependent kinase 9 (CDK9) activity [128]. These resilient cells often exhibit a repressed chromatin state characterized by the methylation of histone H3 lysines 9 and 27 (H3K9 and H3K27) [129]. Heterochromatinization, without recurrent genetic changes, is indeed a distinctive feature of dormant breast cancer cells surviving endocrine therapy. In this case, a stochastic subset of cells transits into dormancy via epigenetic reprogramming. Later, individual cell clusters with divergent phenotypes awaken unpredictably, causing clinical relapse. Inhibitors against G9a-dependent H3K9me2 severely impaired the formation of therapy-induced dormant cells [130]. Surviving AML cells exploit similar senescence and embryonic diapause transcriptional programs to withstand chemotherapy [131]. These adaptive states were associated with downregulation of *MYC* and stem cell genes, and were transient in nature. Entry into senescence-like phenotypes was favored by ATR activity, whereas the egress from resilient phenotypes was associated with increased stem cell potential and leukemia repopulation [131]. Both cell-extrinsic and intrinsic factors drive leukemia persistence after chemotherapy. Adrenomedullin and its cognate receptor CALCRL have been shown to promote cell cycle, DNA repair, and Bcl-2 expression in drug-tolerant AML cells [132]. Likewise, high intrinsic OxPHOS activity mediates cytarabine resistance in AML. In vitro and in vivo targeting of mitochondrial protein synthesis, electron transport chain, or fatty-acid oxidation impaired OxPHOS and enhanced the antileukemic effects of antimetabolites [133].

Whether diapause-like transcriptional programs facilitate the emergence of genes-first or phenotypes-first resistance mechanisms remains undefined. However, the enhanced stem cell signature after transitioning into diapause-like states [131] suggests that intermediate resilience programs might prime leukemic cells for subsequent acquisition of full phenotypic resistance.

## Additional mechanisms favoring phenotypic adaptation: integrated stress response and epigenetic modulation

### Integrated stress response

The integrated stress response (ISR) is a complex signaling network that responds to proteostasis alterations by regulating protein synthesis rate. Besides playing a key role in cell differentiation and immunity, ISR is often co-opted by cancer cells to resist oncogenic stress and pharmacological pressure. The activation of the eiF2/ATF4 branch of the ISR facilitates cell plasticity and is essential for the emergence of therapy-resistant cell states in solid tumors [134, 135]. Moreover, the heme-regulated inhibitor (HRI) kinase functions as a sensor for cytosolic cytochrome c, further engaging the ISR and transactivating ATF4, which contributes to cancer cell persistence under BH3 mimetics [136]. AML blasts exposed to daunorubicine activate an ATF4-mediated, ISR-like transcriptional program that converges to the induction of ABCB1, a drug efflux pump driving chemoresistance [137]. Moreover, ATF4 and ZBTB1 are required to enable cell survival under the nutrient stress triggered by L-asparaginase, a compound widely used in the clinic to deplete serum asparagine and treat ALL. In this context, loss of ATF4 or ZBTB1 sensitizes therapy-resistant T-ALL cells to L-asparaginase [138].

### Modulation of the epigenetic machinery

Chromatin remodeling complexes, such as SWI/SNF and NuRD, play a pivotal role in orchestrating transcriptional plasticity in cancer. In hematologic malignancies, mutations in subunits like ARID1A or CHD4 have been linked to stem-like transcriptional states and adaptive drug resistance [139]. Moreover, H3K27me3-modifying enzymes (e.g., EZH2) and histone demethylases (e.g., KDM6A) dynamically reshape chromatin accessibility to support transcriptional programs that sustain minimal residual disease and promote relapse [139]. Epigenetic modulators can reprogram aberrant chromatin landscapes and transcriptional states. BET inhibitors, such as JQ1 and CPI-0610, displace BRD4 from chromatin and downregulate MYC-dependent transcriptional programs, reversing adaptive survival mechanisms in hematologic malignancies and enhancing antitumor immunity [140, 141]. LSD1 inhibitors block demethylation of H3K4me1/2, stabilizing enhancer landscapes and reversing aberrant differentiation blocks in AML. These agents promote terminal differentiation and sensitize leukemia cells to chemotherapies [142]. HDAC inhibitors, including vorinostat and romidepsin, restore chromatin accessibility and transcriptional activation of pro-apoptotic and differentiation genes [143, 144]. Collectively, these agents modulate chromatin regulators that underpin phenotypic plasticity, making them promising tools to either prevent the emergence of resistant states or reprogram them into therapy-responsive phenotypes.

## A three-step perspective on tackling phenotypes-first resistance mechanisms

**Fig. 7:**
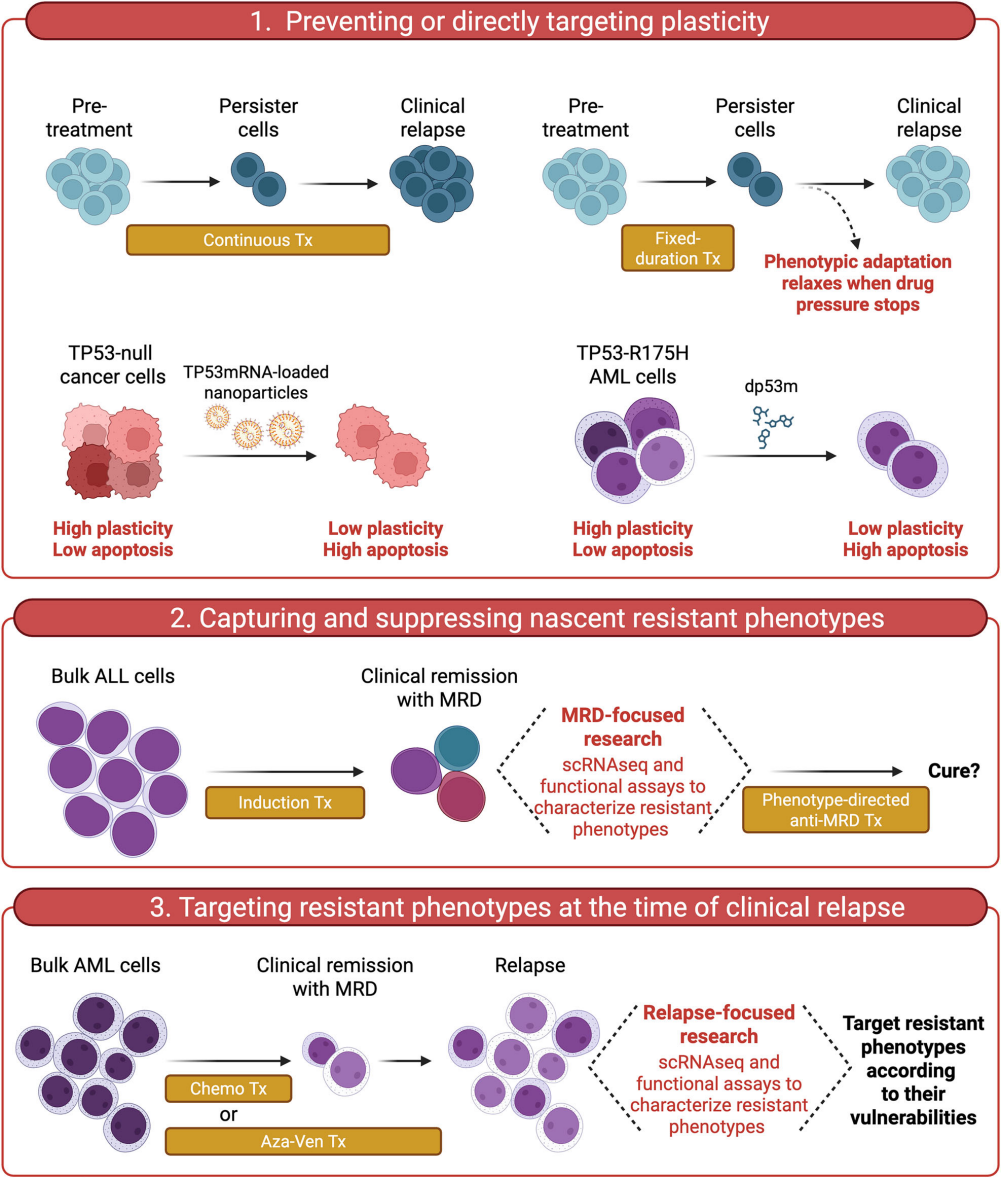
Three-step perspective to counteract phenotypic resistance. **(1) Preventing or directly targeting plasticity**. *Upper panel*. Difference between continuous (left) and fixed-duration (right) treatment in terms of onset of phenotypic adaptation. Fixed-duration treatment enables phenotypic adaptation to relax when drug pressure stops (ref. [15]). *Lower-left panel*. Restoration of TP53 functionality through mRNA-loaded lipid nanoparticles in solid tumors diminishes cell plasticity and fosters apoptosis (refs. [150, 151]). *Lower-right panel*. dp53m dampens gain-of-function activity of TP53-R175H in AML, suppresses cell plasticity, and favors apoptotic response (ref. [154]). **(2) Capturing and suppressing nascent resistance phenotypes**. This approach, mainly explored in the acute lymphoblastic leukemia (ALL) setting, needs robust methodologies to detect and interrogate residual cells that survive induction treatment. Single-cell RNA sequencing (scRNAseq) and additional functional assays may inform cell programs that support ALL persistence, suggesting therapeutic strategies to eradicate residual disease (refs. [40, 156]). MRD minimal residual disease, Tx treatment. **(3) Targeting resistance phenotypes at the time of clinical relapse**. Dissecting both genetic and non-genetic resistance mechanisms through single-cell approaches at clinical progression may help design rational next-line treatments (refs. [36, 78, 103, 157–159]). AML acute myeloid leukemia, MRD minimal residual disease, Aza-Ven azacitidine plus venetoclax.

Having recognized phenotypes-first paths as drivers of treatment resistance, the next challenge is designing novel therapeutic modalities that either prevent their emergence, counteract their expansion when they are just growing, or target their specific vulnerabilities once they are fully established (Fig. 7).

### Strategies to prevent the emergence of phenotypic resistance

Two general principles may be considered to prevent the emergence of phenotypes-first adaptations. First, applying fixed-duration combination regimens that simultaneously target complementary pro-survival pathways. Second, targeting the molecular aberration responsible for the emergence of phenotypic plasticity [145]. The first strategy has already entered clinical practice. In CLL, the combination of ibrutinib plus venetoclax allows the simultaneous targeting of the B-cell receptor signaling pathway and mitochondrial apoptosis [146, 147]. While ibrutinib inhibits pro-survival signals in the lymph node microenvironment and causes leukemic cells to leave the tissue niches, venetoclax antagonizes Bcl-2 and activates mitochondrial apoptosis in the bloodstream, where CLL cells are deprived of the short-lived anti-apoptotic molecules (e.g., Mcl-1) [148]. This combination leads to complete remission in 55% of patients and, in light of the depth of clinical responses, configures a fixed-duration regimen that allows for treatment discontinuation after 15 months. Of note, patients experiencing disease progression after treatment completion were successfully re-challenged with ibrutinib, with an overall response rate of 86% [149]. This suggests that resistant phenotypic states evanish when the therapeutic pressure stops after fixed-duration treatment and when synergism between the two drugs is strong enough to provoke a sharp drop of the leukemic burden.

Focusing on the molecular aberrations responsible for cell plasticity, new technological platforms have been developed to replace wild-type TP53 or target its plasticity-generating mutant forms. Kong and colleagues encapsulated *TP53* mRNA into redox-responsive nanoparticles for selective messenger delivery into hypoxic tumor cells. Exposing cancer cells to *TP53* mRNA-loaded nanoparticles delayed tumor growth and triggered apoptosis in *TP53*^*null*^ hepatocellular carcinoma and non-small cell lung cancer [150]. Similarly, systemic administration of wild-type *TP53* via tumor-targeted nanocomplex prolonged the anti-tumor effect of temozolomide and limited the acquisition of resistance in orthotopic models of glioblastoma multiforme [151]. A selective proteolysis-targeting chimera has been developed for the *TP53* hotspot R175H [152], one of the most frequent TP53 aberrations even in the AML setting [153]. A selective TP53-R175H degrader, dp53m, induced the ubiquitin-proteasome-dependent degradation of the mutant protein while sparing the wild-type counterpart [154]. Moreover, it promoted apoptosis in TP53-R175H-driven cancers [154]. It remains to be explored whether this strategy could be pursued to sensitize *TP53* mutant AML cells to standard chemotherapy or Bcl-2 antagonists. Given the pathogenetic role of mutant *TP53* in AML, we reason that similar strategies could reduce the phenotypic plasticity and decrease the adaptive potential of AML cells to a broad range of therapeutics. As an alternative approach, a bispecific antibody targeting the R175H neoantigen in complex with HLA-A on the cell surface has been generated [155]. This antibody brings CD3^+^ T-cells close to *TP53* mutant cells, thus causing cancer cell apoptosis in animal models.

### Strategies to counteract the expansion of emergent phenotypic resistance

Counteracting the expansion of tumor-beneficial cell states that emerge right after the initiation of treatment and prior to overt disease progression could hold promise to eradicate residual disease. However, this needs a paradigm shift in the research programs, which should focus more thoroughly on the epigenetic and phenotypic aspects of rare cell populations that form minimal residual disease (MRD). In BCR-ABL1^+^ B-ALL, rare persisting cells have the transcriptional features of stemness, long-term dormancy, and drug resistance [156]. Three expression programs have been identified in MRD cells: a pre-BCR signaling program, a stress/autophagy program, and an inflammatory program [40]. Targeting these pathways with RNA-seq-informed chemical inhibitors has proven effective in combination with BCR-ABL1 inhibitors in eradicating residual disease in PDX models. Therefore, MRD cells may be successfully targeted according to the specific transcriptomic/phenotypic state that governs their persistence during clinical remission.

### Strategies to counteract fully established phenotypic resistance

In AML patients overtly progressing on azacytidine and venetoclax, a phenotypic shift towards monocytic and megakaryocytic progenitors has been demonstrated [78, 157, 158]. Importantly, single-cell transcriptomics and metabolic studies have pointed out that venetoclax-resistant monocytic cells are particularly reliant on purine metabolism and, hence, highly sensitive ex vivo to drugs inhibiting one-carbon metabolism or purine-based DNA/RNA synthesis, such as methotrexate and cladribine, respectively [78]. By contrast, megakaryocytic-skewed relapsed AML cells acquire functional Bcl-xL dependency that can be hit by A1331852 [103]. Thus, the developmental direction taken by plastic leukemic cells correlates with specific vulnerabilities and eventually informs effective salvage treatments. Similar approaches are being tested in lymphoid malignancies, where transcriptional OXPHOS^high^ states are often responsible for clinical relapse on BTK inhibitors and potentially sensitive to direct inhibition of ETC complex I [36, 159].

Despite these efforts, several hurdles still limit the clinical applicability of targeting phenotypic adaptations. Table 4 summarizes what we consider the most relevant translational barriers and provides some examples of how pharmacological research has been trying to overcome them [15, 116, 150, 160–162].

**Table 4:** Translational barriers to developing therapeutic strategies against non-genetic adaptation.

Translational barriers	
Heterogeneity-related barriers	Non-genetic, phenotypic adaptations are heterogeneous. They can differ across patients treated with the same drug.
Diagnostic research-related barriers	Identification of phenotypic adaptations often requires multiple unstandardized wet lab techniques.
	Persister cells are few and often hidden in tissue niches with low accessibility
	Functional assays required to identify phenotypic adaptations could be subjected to lower reproducibility compared to genetic testing.
	Expensive single-cell transcriptomic approaches are often required to capture non-genetic adaptive mechanisms. Indeed, multiple escape mechanisms can be detected in individual patients.
	Extensive bioinformatic expertise is required to identify transcriptomic-based resilience programs.
Cell biology-related barriers	Cell programs conferring phenotypic adaptations can play crucial role in homeostasis. Thus, their targeting can result in excessive toxicity. One such example is OxPHOS targeting (ref. [116]).
	Autophagy and integrated stress response, two evolutionarily conserved cell programs conferring phenotypic adaptations, are redundant in nature. Targeting one single protein might not be sufficient to suppress the whole pathway.
Therapeutic research-related barriers	Plasticity and emergence of phenotypic adaptation can be due to something that is lacking, such as wild-type TP53. Delivering something that is lacking is traditionally harder than inhibiting a target that is already in the cell. mRNA therapeutics may hold promise to overcome this issue.
	Anti-apoptotic proteins often upregulated in persister cells, such as Mcl-1 and Bcl-xL, play important role in cardiac and platelet homeostasis. This poses significant challenging in their targeting.
	Non-genetic adaptation is often due to the activity of critical transcription factors. Unlike kinases, surface receptors and anti-apoptotic proteins, transcription factors have long been considered undruggable. Molecular glues may override this limitation in the next future.

See text for references.

## Open questions and concluding remarks

As mechanisms for therapeutic escape unfold, different aspects of cell biology come into play. On the one hand, Darwinian expansion of pre-existing, genetically defined subclones emerges under therapeutic pressure. On the other hand, non-genetic, phenotypes-first adaptation arises as well, compromising treatment efficacy. Given the dynamic and multilayered nature of phenotypic adaptation, which includes microenvironmental changes, different signaling modalities, and transcriptional reprogramming, its specific detection and targeting remain challenging. Among the open themes, one relates to preventing versus chasing resistance mechanisms. Much effort is being placed on identifying resistant pathways to each newly approved drug. Still, little has been made to understand the core reasons behind resistance, i.e., how plasticity develops and how we could target it to prevent drug adaptation. Moreover, additional studies in the specific setting of rapidly adapting hematological malignancies are needed to elucidate the relative contribution of genes-first and phenotypes-first resistance mechanisms. Lastly, a fundamental question related to phenotypes-first mechanisms is whether they emerge from the selection of a pre-existing phenotypic repertoire or rather arise de novo after treatment initiation due to cancer cell plasticity. Cutting-edge technologies and multidisciplinary expertise will be needed to answer these points and provide novel clues to combat or prevent therapy resistance.
